# A method for measuring spatial resolution based on clinical chest CT sequence images

**DOI:** 10.1002/acm2.70078

**Published:** 2025-03-18

**Authors:** Ying Liu, Jingying Shen, Haowei Zhang, Haikuan Liu

**Affiliations:** ^1^ School of Health Science and Engineering University of Shanghai for Science and Technology Shanghai China; ^2^ Institute of Radiation Medicine Fudan University Shanghai China

**Keywords:** CT sequence image, modulation transfer function, spatial resolution

## Abstract

**Purpose:**

This study aimed to develop and validate a method for characterizing the spatial resolution of clinical chest computed tomography (CT) sequence images.

**Methods:**

An algorithm for characterizing spatial resolution based on clinical chest CT sequence images was developed in Matlab (2021b). The algorithm was validated using CT sequence images from a custom‐made chest automatic tube current modulation (ATCM) phantom and clinically reconstructed chest CT sequence images. A region of interest (ROI) was automatically established at the edges of CT image subject to calculate the edge spread function (ESF). The ESF curves from consecutive CT images within the same sequence were fitted into a curve, and the line spread function (LSF) was derived through differentiation. A Fourier transformation of the LSF curve was conducted to obtain the modulation transfer function (MTF). The method's effectiveness was verified by comparing the 50% MTF and 10% MTF values with those calculated using IndoQCT (22a) software. The method was also applied to clinical CT images to calculate MTF values for various reconstructions, confirming its sensitivity by determining spatial resolution of clinically reconstructed images.

**Results:**

Validation experiments based on the phantom CT sequence images demonstrated that the MTF values calculated using the proposed method had an average difference of within ± 5% compared to the results obtained with IndoQCT. Validation experiments with clinical CT sequence images indicated that the method effectively reflects differences and variations in spatial resolution of images under different reconstruction kernels, with the MTF values for B10f‐B50f and D10f‐D50f exhibiting a consistent increase.

**Conclusion:**

A method for measuring spatial resolution using clinical chest CT sequence images was developed. This method provides a direct means of spatial resolution characterization for clinical CT datasets and a more accurate representation of CT imaging quality, effectively reflects variations across different reconstruction convolution kernels, demonstrating its sensitivity.

## 1 INTRODUCTION

Computed tomography (CT) produces detailed images of internal human body structures, providing physicians with precise information for diagnosing various diseases and aiding in the development of treatment plans. CT has become an essential diagnostic tool in routine clinical practice.^1^, ^2^ However, the ionizing radiation inherent in CT scanning poses a risk, particularly when cumulative exposure from repeated scans is considered, as it may elevate the risk of neoplastic transformation. Therefore, it is essential to optimize radiation dose while ensuring adequate CT image quality. In addition, to maximize the clinical utility of CT imaging, an understanding and characterization of image quality parameters are imperative.^3^

In clinical practice, the quality of CT images is contingent upon multiple parameters, including spatial resolution^4^ and noise power spectrum.^5^ Spatial resolution serves as a critical metric for assessing the discernibility of adjacent structures within an image. The modulation transfer function (MTF)^6^, ^7^ is a widely used objective measure of spatial resolution and has been widely employed as a quantitative, spatial frequency‐dependent metric for describing the spatial resolution of medical imaging systems. Currently, MTF can be derived from the measurement of the point spread function (PSF),^8^, ^9^ line spread function (LSF),^10^ or edge spread function (ESF).^11^, ^12^

The spatial resolution characterization of CT images is typically based on static, morphologically uniform phantoms.^9^, ^13^, ^14^, ^15^, ^16^ Nofrianto et al.^9^ compared the differences in MTF measurements on GE phantom CT images using two different software tools, IndoQCT and ImQuest. This study measured MTF values on GE phantom CT images under different slice thicknesses and reconstruction filters. Elzami et al.^14^ proposed an improved MTF measurement method using a phantom made of polymethyl methacrylate (PMMA). In their study, the median pixel value of the phantom CT images was used to replace all pixel values within the image, effectively homogenizing the phantom CT images. Then, the ESF curve was measured, the LSF curve was calculated, and the MTF curve was obtained through Fourier transformation. Anam et al.^16^ developed a new automatic MTF measurement algorithm for the existing ACR 464 phantom and evaluated the impact of reconstruction filters and field of view on CT image spatial resolution. Phantoms are widely used in CT imaging research and device calibration as substitutes for clinical CT images. Compared to clinical CT images, phantoms can withstand high‐dose radiation exposure and are not affected by anatomical structures or individual patient differences during the research process. However, phantoms are objects designed with precise geometries and known physical properties according to research needs. They are static and uniform. In contrast, clinical CT images contain the complex anatomical structures of real patients, with significant variations in anatomical morphology, organ size, and density distribution across individuals. Additionally, clinical CT images may include motion artifacts and high noise levels due to imaging conditions. But phantoms are imaged in controlled environments, with minimal patient‐related artifacts or motion effects, and generally exhibit lower noise levels. Therefore, phantom CT images lack clinical relevance and cannot fully reflect the information in clinical CT images, meaning their spatial resolution cannot effectively represent the spatial resolution of clinical CT images.

Measuring the spatial resolution of clinical CT images would provide a more accurate quantification of true CT image quality. Jeukens et al.^1^ proposed a proof‐of‐concept computer algorithm that uses CT images of the abdominal venous phase in the human body to calculate image quality metrics such as noise, spatial resolution, and contrast. They also assessed the consistency between the objective image quality metrics and subjective ratings from radiologists, achieving good results. Sanders et al.^13^ developed an automated technique for evaluating the spatial resolution features of clinical CT images, focusing on CT images of the human chest and pelvis. This method uses multi‐threshold techniques to segment the patient from surrounding objects, constructs a grid of the patient using tetrahedral elements, and generates a 3D view. They then performed ESF measurements on the patient's skin, differentiated them to obtain the LSF, and calculated the Fourier transform of the LSF to obtain a CT spatial resolution index similar to MTF, yielding promising results. Most existing methods for measuring the spatial resolution of clinical CT images are based on a single CT image, from which the overall spatial resolution is calculated. However, a single CT image can only reflect the spatial resolution of a localized area and cannot comprehensively assess the resolution distribution across the entire three‐dimensional space. Additionally, single CT images may contain noise or artifacts, factors that can lead to unstable measurement results and reduced accuracy in spatial resolution calculations.

This study aimed to develop an MTF measurement method based on clinical chest CT sequence images to characterize the spatial resolution of these images. The method was validated using CT sequence images from a custom‐made chest ATCM phantom and clinical chest CT sequence images reconstructed with different kernel sizes. The main contributions of this study are summarized as follows:

1. A new MTF measurement method based on clinical chest CT sequence images was proposed. Sequential images took advantage of the three‐dimensional nature of CT imaging, allowing for the analysis of resolution across different planes. This effectively addressed the limitations of single CT image measurement methods in describing overall spatial resolution, improved the stability of spatial resolution measurements, and enhanced the accuracy of spatial resolution measurement results.
2. The application of this MTF measurement method characterized spatial resolution in clinical chest CT sequence images, exhibiting outstanding performance. The MTF measurement method developed in this study was applicable to real clinical scenarios and contributed to advancing technology in the field of medical image evaluation, as well as promoting the widespread clinical application of these techniques.

The remainder of this study is organized as follows: Section 2 provides a detailed description of the proposed method, along with an explanation of the phantom used in the experiments and the clinical CT sequence image dataset reconstructed with various kernel sizes. Section 3 presents the experimental results obtained from validating the method using both the phantom and clinical CT sequence images. Finally, Sections 4 and 5 discuss the findings and offer conclusions regarding the research content.

## 2 MATERIALS AND METHODS

### 2.1 MTF measurement

**FIGURE 1:**
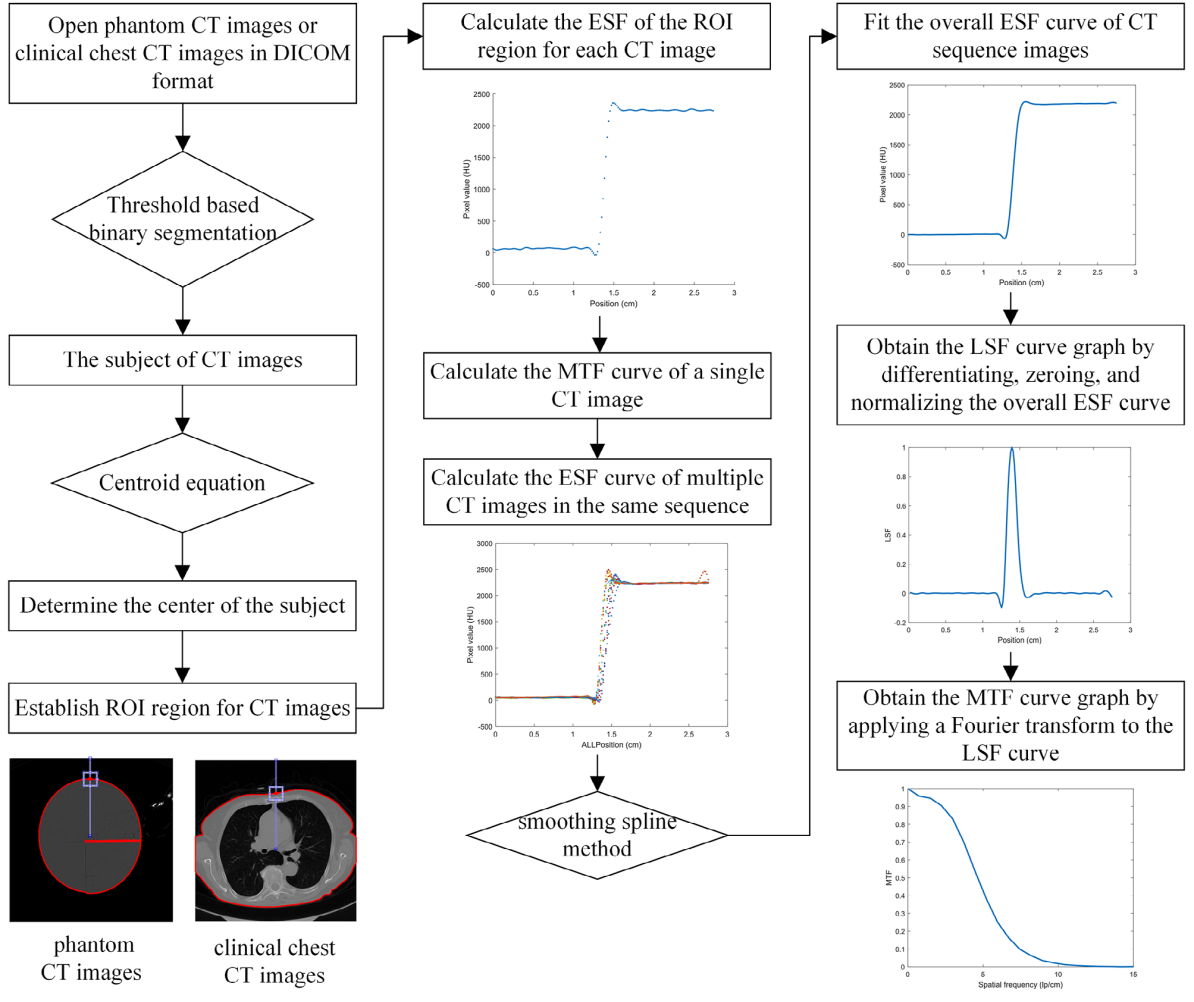
Measurement process of MTF.

MTF measurement was implemented in Matlab (2021b), and the specific algorithm flowchart was shown in Figure 1. The CT images were opened in their original DICOM format, and threshold‐based binarization was performed to segment the main object in the image. The centroid (*x*_a_, *y*_b_) of the object was calculated using Formula 1, where *N* was the total number of pixels in the object region, and (*x*_n_, *y*_n_) represented the coordinates of each pixel.

(1) $$\left(x_{a},y_{b}\right)=\left(\frac{1}{N}\sum_{n=1}^{N}x_{n},\frac{1}{N}\sum_{n=1}^{N}y_{n}\right)$$

A vertical line was drawn from the object's center to the top of the CT image, and the intersection of this line with the upper boundary of the object was used as the center, with coordinates (*x*_c_, *y*_c_). The region of interest (ROI) was defined by extending from *x*_c_ ‐ 20 pixels to *x*_c_ + 20 pixels and from *y*_c_ ‐ 20 pixels to *y*_c_ + 20 pixels. The ESF for this ROI was computed by averaging the CT values across each pixel column within the region, where the x‐axis represented the pixel position and the y‐axis represented the average CT value. To ensure a smoother curve and improve the spatial resolution of the derived MTF, spline interpolation was applied, adding four interpolated data points between each pair of original pixel values. The ESF curve was subsequently differentiated and Fourier transformed to produce the MTF curve for a single CT image, which represents the method for measuring MTF based on a single CT image. To generate the overall MTF curve, these steps were repeated for consecutive CT images in the same sequence.

The ESF results from the CT sequence images were fitted using the smoothing spline method, resulting in an overall ESF curve for the entire CT sequence. In this curve, the x‐axis represented the pixel positions, and the y‐axis represented the average CT values within the ROI across the sequence images.

The ESF curve was differentiated to produce the LSF curve, which was then processed with zeroing and normalization to enhance stability and mitigate the influence of noise on MTF calculation. The zeroing adjustment involved subtracting the average of the first five points from the entire curve to ensure that the leftmost value of the LSF curve equaled zero. Normalization was applied by scaling the curve with the range of its maximum and minimum values, resulting in an MTF curve with an amplitude normalized to a maximum value of 1. The MTF curve was obtained by applying a Fourier transform to the LSF curve. Given the additional noise artifacts present in clinical CT images, additional steps were implemented beyond fitting the ESF curve and performing zeroing and normalization on the LSF curve. Specifically, the MTF curve was further refined using Formula 2 to enhance stability, with the fitting coefficients a, b, c, and d automatically optimized through the fit function for best alignment with the data.

(2) MTFfit(f)=cexp−π2f2/d+a1+4π2f21+4π2f2b2b2c+a

The spatial frequency at an MTF value of 50% is considered the spatial resolution of the CT image sequence, reflecting the system's capacity to discern details at moderate contrast levels. The spatial frequency at the MTF value of 10% denotes the system's capability to resolve details at low contrast.

### 2.2 Validation of phantom CT images

**FIGURE 2:**
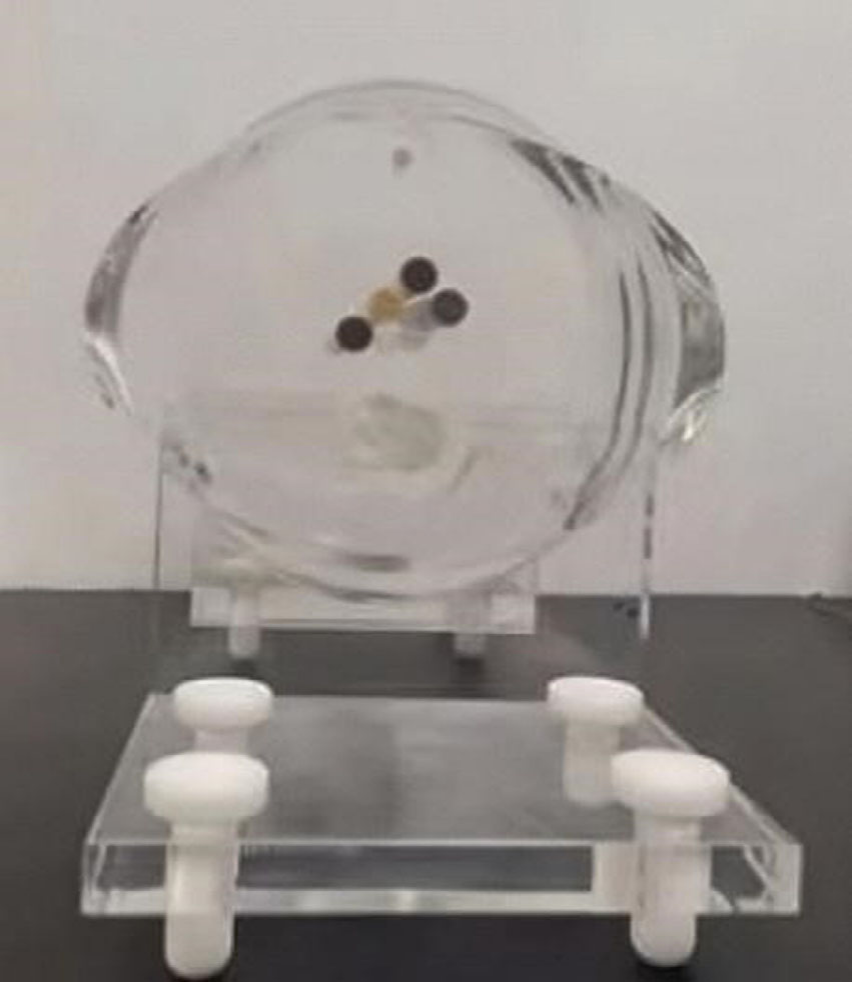
The custom‐made chest ATCM phantom.

This study employed a novel chest automatic tube current modulation (ATCM) phantom.^17^, ^18^, ^19^ The phantom was designed based on simulation results of the equivalent attenuation for each 1 cm slice of the computational phantom representing the Chinese reference man, and was constructed from polymethyl methacrylate (PMMA). The phantom features clearly defined edges, enabling precise calculation of ESF and MTF. The physical representation of the phantom is illustrated in Figure 2. The CT images obtained from this custom‐made phantom were captured using the United Imaging UIHuCT960+ CT scanner (United Imaging Healthcare, China), with the default scanning parameters under the ATCM system outlined in Table 1.

**TABLE 1:** Default scan parameters.

Scan parameters	Default values
Tube voltage	120 kVp
Rotation time	0.5s
Collimation width	40 mm
Modulation level	L15
Pitch	1.0
Reconstruction kernel	B
Reconstruction slice thickness	5 mm

To validate the accuracy of the spatial resolution results derived from the proposed method on the phantom CT images, this study utilized IndoQCT software (version 22a)^16^ to compute the spatial resolution of the phantom images. The results were then compared to those obtained from the proposed method. Developed by Choirul Anam and colleagues at Diponegoro University, IndoQCT facilitates the quality assessment of individual CT images from various ACR phantoms, including measurements of spatial resolution among other parameters. Since the IndoQCT software calculates spatial resolution only for individual CT images, the overall spatial resolution is represented by the average and standard deviation from a series of consecutive CT images for comparison.

### 2.3 Validation of clinical CT images

This study utilized clinical chest CT sequence images acquired from a volunteer, using the SIEMENS SOMATOM Definition Edge scanner. This study was reviewed and approved by Shanghai Zhongye Hospital, with approval reference number Zhongye Lunshen(2024) LS0001.

**FIGURE 3:**
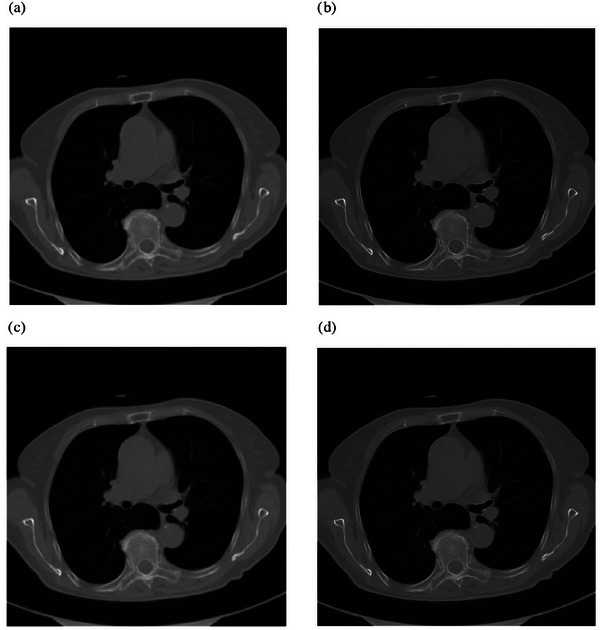
Clinical chest CT images after reconstruction.

To assess the sensitivity of the spatial resolution results obtained from the proposed method on clinical CT sequence images, this study reconstructed the images using various types and sharpness levels of convolution kernels. Specifically, clinical chest CT images from the same sequence were reconstructed with two different types of kernels: the body convolution kernel B, categorized into five groups (B10f, B20f, B30f, B40f, and B50f), and the dual‐energy convolution kernel D, categorized into five groups (D10f, D20f, D30f, D40f, and D50f). Each image measured 512×512 pixels, with a slice thickness of 5 mm. Taking the 25th CT image as an example, Figure 3 shows the results reconstructed by four different kernels.

## 3 RESULTS

### 3.1 Validation of phantom CT images

**FIGURE 4:**
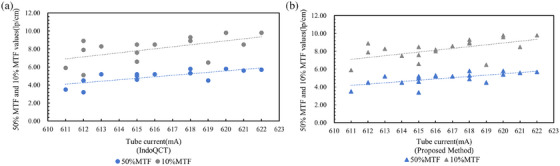
Trends in the spatial resolution values of single CT images. (a) IndoQCT. (b) The method based on a single CT image.

The CT sequence obtained by scanning the phantom under the default parameters of the ATCM system includes 40 CT images. For analysis, the images from the 16th to the 35th were selected. The 50%MTF and 10%MTF values calculated using the method for measuring MTF based on a single CT image, were compared with those obtained using IndoQCT software. In this study, the ROI size for the MTF calculations was 41×41 pixels, and no processing was applied to the ESF curves. To ensure a fair comparison, the same ROI size of 41×41 pixels was used in the IndoQCT analysis without processing the ESF curves. The comparative 50%MTF and 10%MTF values are detailed in Table 2, organized by tube current. Figure 4 depicts the trend of spatial resolution values as a function of tube current for both methods.

**TABLE 2:** Spatial resolution of single CT images.

		IndoQCT		Sigle CT image	
Number	Tube current (mA)	50%MTF (lp/cm)	10%MTF (lp/cm)	50%MTF (lp/cm)	10%MTF (lp/cm)
1	611	3.5	5.9	3.36	6.17
2	612	4.5	7.9	4.21	7.67
3	612	4.5	8.9	4.16	7.89
4	613	5.2	8.3	4.67	8.66
5	614	4.5	7.5	4.1	7.55
6	615	3.4	6.6	3.52	6.46
7	615	4.6	7.6	4.33	7.91
8	615	5.0	6.6	4.37	7.95
9	615	5.2	8.5	4.83	8.79
10	616	5.2	8.0	4.72	8.57
11	616	5.3	8.2	4.77	8.93
12	617	5.2	8.6	3.96	7.34
13	618	4.9	9.0	3.8	7.13
14	618	5.3	8.9	5.27	9.54
15	618	5.8	9.3	4.74	8.67
16	619	4.5	6.5	3.99	7.28
17	620	5.4	9.6	5.34	9.66
18	620	5.8	9.8	5.38	9.73
19	621	5.6	8.5	5.07	9.18
20	622	5.7	9.8	5.14	9.3

Table 2 reveals that the 50%MTF and 10%MTF values for single CT images calculated by both methods exhibit considerable fluctuations and discrepancies within the same sequence, with an average difference of 9.9%. Additionally, Figure 4 illustrates a positive correlation between the spatial resolution values derived from both methods and the tube current.

**FIGURE 5:**
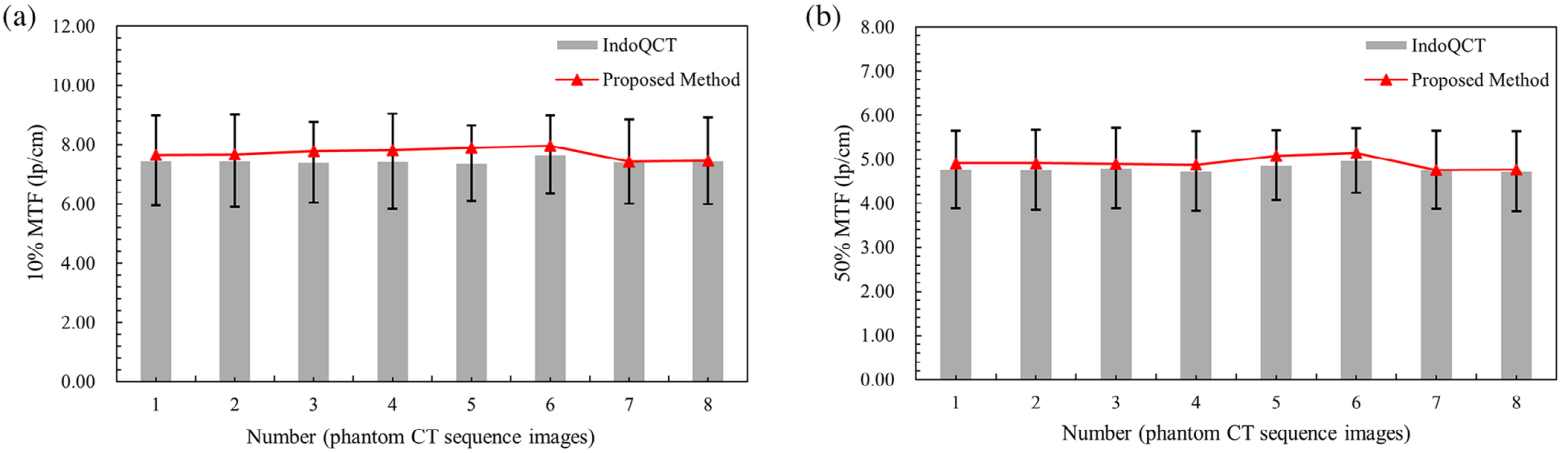
Comparison of MTF values of different methods.

Under different ATCM system parameters, eight sets of CT sequence images were scanned, each sequence consisting of 40 CT images. The scanning parameters for each group were shown in Table 3, and any unspecified parameters were identical to the default scanning settings. The 15th to 30th images in each sequence were analyzed to calculate the spatial resolution at 50% MTF and 10% MTF using two methods, expressed in line pairs per centimeter (lp/cm). Figure 5, respectively, displays the error bar charts for the 50% MTF and 10% MTF values obtained through the IndoQCT, with red points indicating the results from the proposed method. The results calculated using this method for all eight sequence image sets were within the error margins of the software method, with differences from the software's average values remaining within ± 5%. Furthermore, the findings reveal that the parameters—tube voltages ranging from 80 to 140 kV, pitch values between 0.8 and 1.4, and collimation widths of 20–80 mm—exert no statistically significant effects on the 50% MTF and 10% MTF, which is consistent with the characteristics of the ATCM system.

**TABLE 3:** Scan parameters.

Number	Tube voltage	Pitch	Collimation width
1	120	1	40
2	100	1	40
3	80	1	40
4	140	1	40
5	120	1	20
6	120	1	80
7	120	0.8	40
8	120	1.4	40

**FIGURE 6:**
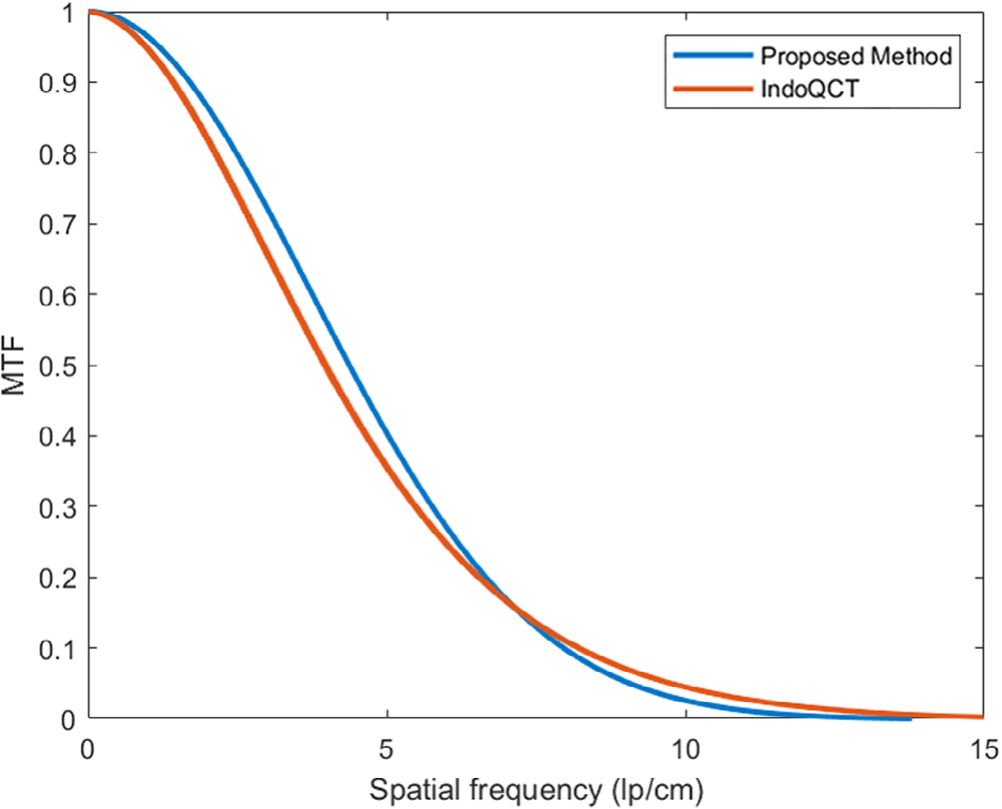
Comparison of MTF curves of different methods.

Furthermore, Figure 6 presents the MTF curves for the CT sequence images derived from both the proposed method and the IndoQCT software, highlighting the comparability between the MTF curves generated by these two approaches.

### 3.2 Validation of clinical CT images

**FIGURE 7:**
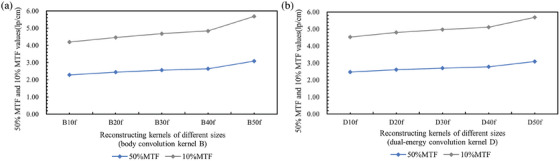
Trends of spatial resolution values.

**FIGURE 8:**
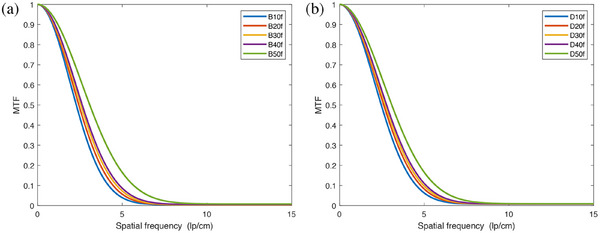
MTF curves of clinical chest CT sequence images after reconstruction (a) B convolution kernel, (b) D convolution kernel.

The clinical chest CT sequence images reconstructed with different convolution kernels included 49 CT images, from which the 20th to 38th images were selected for analysis. The spatial resolution results obtained using the proposed method are presented in Table 4. The trends in spatial resolution values for the body convolution kernel group B and the dual‐energy convolution kernel group D are depicted in Figure 7a,b, respectively, while Figure 8 provides a comparison of the MTF curves for both groups. The findings indicated that the proposed method effectively highlights variations in CT images reconstructed with different types and levels of sharpness of convolution kernels. As the values increase from B10f to B50f and from D10f to D50f, the reconstructed images become sharper, resulting in higher values of 50%MTF and 10%MTF.

**TABLE 4:** Spatial resolution of clinical CT sequence images.

	Spatial resolution (lp/cm)	
Number	50%MTF	10%MTF
B10f	2.29	4.19
B20f	2.44	4.46
B30f	2.56	4.68
B40f	2.64	4.84
B50f	3.09	5.68
D10f	2.47	4.53
D20f	2.61	4.80
D30f	2.70	4.97
D40f	2.78	5.11
D50f	3.09	5.69

## 4 DISCUSSION

Characterizing the spatial resolution of CT images is a crucial method for quantifying CT image quality, assessing the capabilities of CT imaging systems, and optimizing clinical protocols. Traditional spatial resolution measurements using phantom CT images have inherent limitations. Phantoms are static, homogeneous objects and do not account for factors such as patient respiratory motion or scan variability encountered in clinical imaging. As a result, phantom CT images do not fully represent clinical CT images. This study introduces a new spatial resolution measurement method based on clinical chest CT sequence images, which can consistently and efficiently calculate spatial resolution values in both custom‐made chest ATCM phantom CT sequence images and clinical chest CT sequence images.

The ATCM system is an automatic tube current modulation technique used in CT scanning that modulates the x‐ray tube current automatically to maintain image quality for clinical diagnosis while minimizing the radiation dose received by the patient. Data presented in Table 2 show that the Pearson correlation coefficients for the 50% MTF and 10% MTF values between the IndoQCT method and the proposed method are 0.86 and 0.75, respectively, indicating a strong positive correlation between the two methods. The correlation coefficients between tube current and the 50% MTF and 10% MTF values, as calculated by the two methods, are 0.67, 0.56, 0.61, and 0.58, respectively. This suggests a moderate positive correlation between tube current and spatial resolution within a certain range of CT images from a single scan using the ATCM system. As the tube current increases within this range, spatial resolution also tends to improve. Furthermore, as shown in Figure 4, the spatial resolution of individual CT images exhibits significant fluctuations, indicating some instability. Compared to existing methods based on single CT images, the MTF measurement method proposed in this study, based on CT image sequences, offers the advantage of greater stability. Data presented in Table 3 and Figure 5 demonstrate that across a range of 80–140 kV tube voltage, 0.8–1.4 pitch, and 20–80 mm collimation width, the influence of these parameters on both the 50%MTF and 10%MTF values is statistically insignificant. This suggests that when the ATCM system adjusts the tube current to account for patient size and scan region, variations in tube voltage, pitch, and collimation width within this range do not impact the spatial resolution of the images. These observations are congruent with the research outcomes reported by Dabli et al.^20^ and align with the expected functionality of the ATCM system. As shown in Figure 5, the results of the proposed method lie within the error range of IndoQCT, with deviations from its mean value confined to ± 5%, providing partial evidence for the feasibility of the proposed method.

In CT image reconstruction, the convolution kernel is a specific algorithm that influences image smoothness and sharpness. The experiment used both body convolution kernel B and dual‐energy convolution kernel D, with variations from B10f to B50f and D10f to D50f. An increase in the kernel value correlates with enhanced image sharpness and more defined edges, though this increase in sharpness may also lead to higher image noise. The data in Table 4 show that the method proposed in this study obtained results consistent with the reconstruction trend when calculating the spatial resolution of clinical chest CT sequence images reconstructed with different‐sized convolution kernels: for the B reconstruction kernel, the 50% MTF value increased from 2.29 (B10f) to 3.09 (B50f), and the 10% MTF value increased from 4.19 (B10f) to 5.68 (B50f); for the D reconstruction kernel, the 50% MTF value increased from 2.47 (D10f) to 3.09 (D50f), and the 10% MTF value increased from 4.53 (D10f) to 5.69 (D50f). These data indicate that with an increase in the sharpness of the convolution kernel, spatial resolution is significantly improved, validating that the method proposed in this study has sufficient sensitivity to reflect changes in spatial resolution caused by different reconstruction methods and parameters.

In clinical practice, image spatial resolution plays a crucial role in the detection of subtle anatomical structures and lesions. For example, higher spatial resolution facilitates the accurate identification of small pulmonary nodules or vascular boundaries, thereby improving the diagnostic rate of early lesions. Additionally, the impact of convolution kernels on resolution observed in this study suggests that by optimizing reconstruction parameters, it is possible to effectively manage noise while maintaining adequate resolution. This sensitivity provides a quantitative foundation for different clinical scenarios, such as high‐resolution lung scans and low‐contrast soft tissue evaluations, offering valuable insights for the process optimization of clinical CT image quality characterization.

The approach to characterizing spatial resolution in CT image sequences exhibits certain limitations at present. First, in the experiments, CT images reconstructed with sharper convolution kernels may suffer from excessive noise, which can hinder the accurate acquisition of ESF curves, thereby impacting the spatial resolution results. CT imaging is susceptible to noise and clinical artifacts. For example, quantum noise, common in low‐dose scans, can obscure details of small lesions, while artifacts such as beam hardening and motion artifacts can distort the image, thus affecting diagnostic accuracy. Noise, artifacts, and even patient sweat, hair, and clothing can make the ESF curve less clean and stable, further increasing the uncertainty of spatial resolution calculation. To address this, improving the fitting technique for the ESF curve is a direction for future optimization. Secondly, phantom CT images are uniformly structured, with the boundary between the phantom and air providing a clear edge, which is adequate for characterizing the spatial resolution of CT images. In this study, the spatial resolution was characterized using the boundary between the skin and air in clinical CT images. The application of adaptive filtering in some clinical CT images during reconstruction allows for differential treatment of various edges, such as those of the spine or soft tissues. Therefore, further exploration of ROI selection and spatial resolution characterization based on specific region characteristics could enhance clinical applications. Finally, this study was limited to CT sequence images from a custom‐made chest ATCM phantom and clinical chest CT sequence images. Future clinical applications could expand the dataset to include CT images from different devices and anatomical regions, such as the head and abdomen, to improve the generalizability of the method.

## 5 CONCLUSION

This study developed a spatial resolution measurement method for clinical chest CT sequence images. Experimental results demonstrated that the 50%MTF and 10%MTF values of CT sequence images differed from those obtained by IndoQCT by less than ± 5%, confirming the method's accuracy. Additionally, applying this method to calculate the spatial resolution of clinical CT sequence images reconstructed with different convolution kernel sizes effectively captured the variations in spatial resolution across different kernels, demonstrating sufficient sensitivity. This algorithm enables rapid quantification of spatial resolution during clinical examinations, providing a scientific foundation for selecting reconstruction algorithms and aiding in the optimization of scan protocols, which is of clinical significance. In future studies, this method could serve as a simple tool for routine quality monitoring of CT equipment, contributing to the continuous improvement of CT image quality.

## AUTHOR CONTRIBUTIONS

Conceptualization and study design: Jingying Shen. Data collection: Ying Liu and Haowei Zhang. Statistical analysis and data interpretation: Jingying Shen. Manuscript preparation: Jingying Shen and Haikuan Liu. All authors have read and approved the final manuscript.

## CONFLICT OF INTEREST STATEMENT

The authors declare no conflicts of interest.

## ETHICS STATEMENT

This study was reviewed and approved by Shanghai Zhongye Hospital, with approval reference number Zhongye Lunshen(2024) LS0001.

## Data Availability

Data available on request from the authors. The data that support the findings of this study are available from the corresponding author upon reasonable request.
